# Identifying periods impacted by sewer inflow and infiltration using time series anomaly detection

**DOI:** 10.1016/j.wroa.2024.100278

**Published:** 2024-11-12

**Authors:** Jingyu Ge, Jiuling Li, Ruihong Qiu, Tao Shi, Zi Huang, Yanchen Liu, Zhiguo Yuan

**Affiliations:** a Australian Centre for Water and Environmental Biotechnology (formerly AWMC), The University of Queensland, St. Lucia, Brisbane, 4072, QLD, Australia; b School of Electrical Engineering and Computer Science, The University of Queensland, St. Lucia, Brisbane, 4072, QLD, Australia; c State Key Joint Laboratory of Environment Simulation and Pollution Control, School of Environment, Tsinghua University, Beijing, 100084, PR China; d School of Energy and Environment, City University of Hong Kong, Hong Kong SAR, China; e State Key Laboratory of Marine Pollution, City University of Hong Kong, Hong Kong SAR, China

**Keywords:** Inflow and infiltration, Dry/ wet weather, Data, Time series, Anomaly detection

## Abstract

Accurate diagnosis of sewer inflow and infiltration (I/I) is crucial for ensuring the safe transportation of sewage and the stability of wastewater treatment processes. Identifying periods impacted by I/I is essential for I/I diagnosis, but current methods lack a standard criterion and require adaptation to specific conditions, resulting in low accuracy, complexity, and limited generalizability. This paper proposes a novel approach to distinguish I/I periods from time series of sewer measurements based on anomaly detection theory through an iterative use of a time-series reconstruction model. This method eliminates the need for external data such as rainfalls and avoids intensive manual data analysis. Operating directly on in-sewer data, it enhances accuracy compared to existing approaches and is applicable to various external factors such as rainfall, snowmelt, and seawater intrusion. The method can be applicable to a broad range of monitoring data, including flow rate, temperature, and conductivity. Validated through simulation studies and demonstrated via real-life applications, this method offers an efficient solution for I/I detection, facilitating further I/I diagnosis, including I/I quantification and location identification.

## Highlights

• A novel method to distinguish wet/ dry periods for sewer inflow and infiltration analysis.
• Integrates anomaly detection via time-series reconstruction and iterative algorithm.
• Enhances the accuracy and precision and reduces human-driven pre-analysis.
• Robustly handles inflow and infiltration by factors beyond rainfall, including snowmelt and seawater tides.
• Capable of being applied to various in-sewer measurements.
• Validated through simulation studies and real data application.

## Graphical abstract

**Figure:**
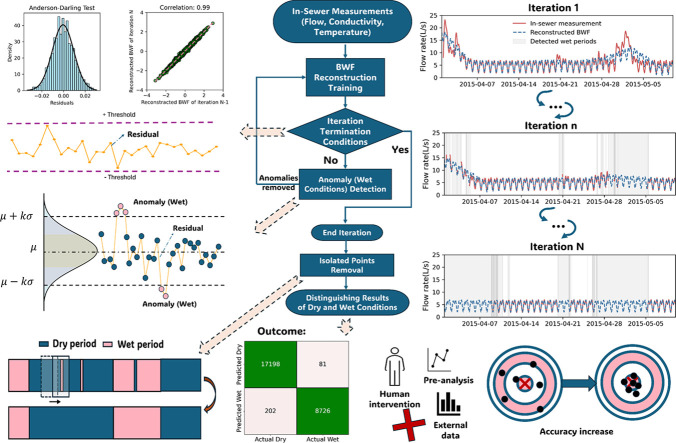

## 1 Introduction

The effective operation of urban sewerage systems is paramount for maintaining urban environments and public health (Rehan et al., 2014). While the base wastewater flow (BWF), generated under dry weather conditions from residential, commercial, and industrial sources, typically displays relatively small variations, the total flow through a network can be elevated to levels several times higher than the BWF (Chen et al., 2014; Li et al., 2019), due to intrusion of external water (Chandler and Lerner, 2015). This is true for both combined sewers and separate sanitary sewers. The external waters, known as inflow and infiltration (I/I), are often a consequence of rainfalls/snowfalls (Panasiuk et al., 2022) or seawater tides (Cahoon and Hanke, 2019). Inflow typically refers to storm or snowmelt water entering the sewer network via misconnections and/or maintenance holes (Chandler and Lerner, 2015). Infiltration is primarily caused by groundwater intrusion via damaged pipes(Karpf and Krebs, 2013), which can be initiated or intensified by rainfall or seawater tides (Cahoon and Hanke, 2019). The presence of I/I poses significant challenges to the system (Yuan, 2019), including flooding (Mohandes et al., 2022), water quality contamination (Ryu et al., 2017; Jiang et al., 2019), infrastructure damage (De Bénédittis and Bertrand-Krajewski, 2005), and complications to wastewater treatment (Ellis and Bertrand-Krajewski, 2010). Therefore, detecting and quantifying I/I has become critical for sewer maintenance and rehabilitation (Zhang and Parolari, 2022; Sowby and Jones, 2022).

The correct identification of periods with and without I/I is important for I/I quantification, location identification and subsequent management (Choi and Schmidt, 2023; Perez et al., 2024; Sydney Water Corporation, 2021; Water Corporation, 2023). Misidentifying periods with I/I as non-I/I periods, or vice versa, can misguide the sewer maintenance program and the operation of sewer networks and wastewater treatment, potentially serious financial and environmental implications. There is currently no standardised criterion for identifying I/I periods. Existing methods primarily distinguish between wet and dry conditions based on rainfall data (Shelton et al., 2011; Perez et al., 2024), as rainfall is often the main trigger for I/I (Zhang et al., 2018a; Zhou et al., 2023). Daily or hourly rainfall thresholds are needed by these methods, above which a period is classified as a wet period (Staufer et al., 2012; Rezaee et al., 2022; Zhang et al., 2018 a; Ge et al., 2024). These thresholds are often set by taking into consideration the regional variations in how systems respond to rainfall (Ge et al., 2024). Factors such as the hydrogeological formation of the topsoil, rock density, slope and soil moisture can influence the selection of these thresholds (Staufer et al., 2012). Even so, these methods often give erroneous results, as the same rainfall incurs I/I on some occasions but not in others due to, e.g., the different soil moisture levels on all occasions. Moreover, rainfall-induced I/I typically experiences varying delays between the rainfall and the time I/I occurs, with a duration influenced by various factors, such as soil moisture level and rainfall intensity (Zhang et al., 2018a; Perez et al., 2024). All these factors increase the complexity of setting fixed thresholds and delay coefficients, resulting in the need for manual intervention and pre-analysis of data (Rezaee et al., 2022; Karpf and Krebs, 2021).

Other I/I contributors, such as snowmelt and seawater tide, also need to be considered in addition to rainfall. Karpf and Krebs (2011) suggested referencing atmospheric temperature to assess snowmelt-induced I/I, with I/I ruled out if temperatures on the day and the three days prior are outside the range of −2 °C to 2 °C. This is only a rough method, resulting in low accuracy. For groundwater infiltration, Ge et al. (2024) used groundwater level data alongside rainfalls to distinguish with and without infiltration, recognising groundwater level data may not always be available.

In summary, existing methods rely on external inputs to identify periods with and without I/I, and often require human intervention in the classification, yet with limited accuracy due to the complex factors influencing the I/I generation process. To address these issues, we propose a new method that identifies periods with and without I/I based on data directly collected from sewers rather than external data such as rainfall, temperature or groundwater level, thus bypassing the need for analysing the I/I generation process and accounting for regional variations. Sewer flow (Choi and Schmidt, 2023; Zhang and Parolari, 2022) and sewage quality parameters such as temperature (Panasiuk et al., 2019; Zhou et al., 2023) and conductivity (Aumond and Joannis,2008; Zhang et al., 2018b) are heavily influenced by I/I. In the absence of I/I in dry conditions, the time series of sewer measurements should display regular patterns. The impacts of I/I are directly reflected as anomalies in the time series, which can be detected using advanced data analytics. Performance is tested using both simulated and real-life data and compared with the currently used methods.

## 2 Results and discussion

### 2.1 Developed method of dry/wet distinguishing

**Fig. 1:**
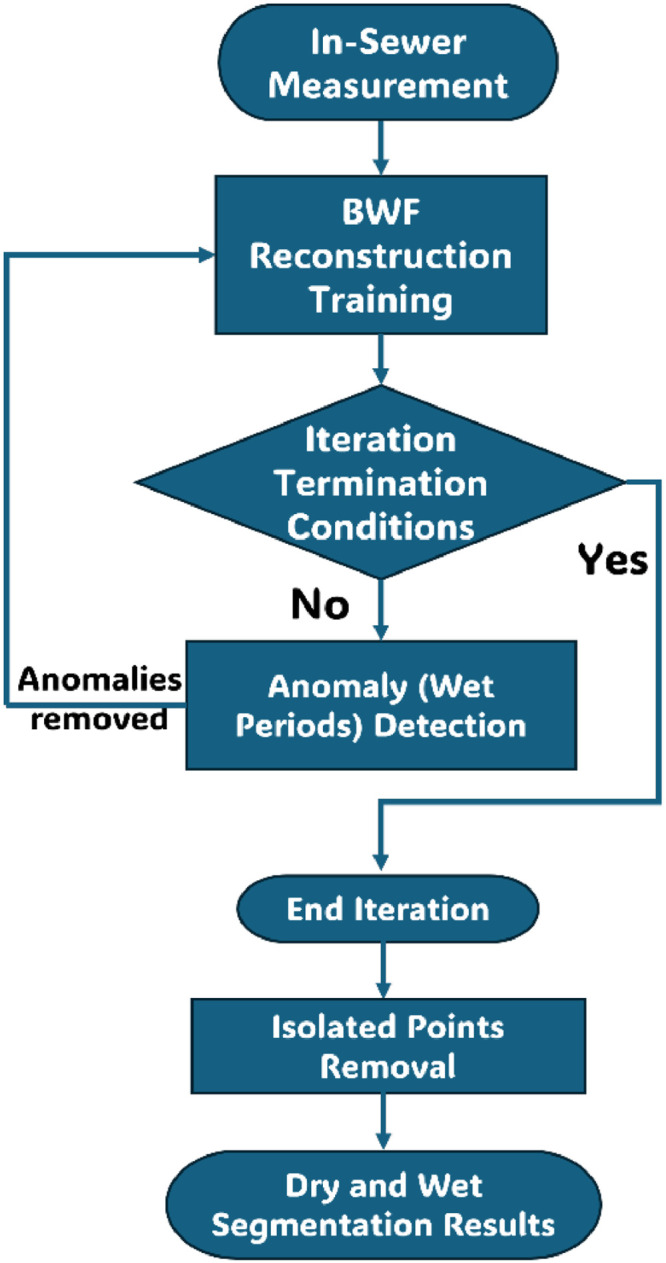
Flow chart of the algorithm.

This method is based on an iterative algorithm comprising four parts (Fig. 1): (1) BWF reconstruction model training, (2) anomaly detection, (3) iteration termination checks, and (4) isolated points removal.

The BWF reconstruction model is to characterise normal states that are not affected by I/I. The existing Prophet model (Taylor and Letham, 2018), originally developed for time series forecasting, especially for financial data, is employed for this purpose. The prophet model is well-suited because of its ability to capture human activity or nature-related patterns in the data, which are precisely the constitutive properties of the sewer monitoring data profile in dry conditions. The model ((1), (2), (3), (4)) describes the in-sewer monitoring data series M(t) (flow or water quality parameters) in four parts, trends Tr(t), periodic variations Pe(t), exceptional events Ev(t) and residual ε(t), selected based on our *a priori* knowledge of human activities and natural variations (Duan et al., 2024). Factors such as population growth and variations in water usage drive the observed long-term trends in the data (Yan et al., 2024). Regular daily activities and seasonal changes lead to periodic fluctuations (Zhang et al., 2018a). Event terms describe the impacts of special events except I/I, such as changes in water usage habits caused by holidays and weekends (Perez et al., 2024), sudden rises in water temperature due to extreme weather, and other irregularities. Short-term fluctuations or noise are captured in the residual component.

(1) M(t)=Tr(t)+Pe(t)+Ev(t)+ε(t)

(2) $$Tr\left(t\right)=\left[\delta_{0}+a{\left(t\right)}^{⊺}\delta \right]t+\left[\gamma_{0}+a{\left(t\right)}^{⊺}\gamma \right]$$

(3) $$Pe\left(t\right)=\sum_{l}\sum_{n=1}^{N_{l}}\left(a_{n,l}\cos(\frac{2\pi nt}{p_{l}}\right)+b_{n,l}\sin\left(\frac{2\pi nt}{p_{l}}\right))$$

(4) ∀t∈D,Ev(t)=κ

where the trend term is represented by head-to-tail linear functions (Eq. 2), the index vector a(t)∈{0,1} is used to indicate whether it is a joint point of linear functions or not, and the initial growth rate and offset are set to be $\delta_{0}$ and $\gamma_{0}$, which are adjusted by δ and γ to regulate the change of growth rate and offset between different linear functions. The periodic variation term is represented by the accumulation of multiple periods, such as daily, monthly, and yearly cycles (Eq. 3). Each periodic cycle is indexed by l, which defines the type of period using $p_{l}$. For example, for hourly data, setting $p_{l}$ equal to 24 corresponds to daily cycles. Every cycle l is represented by a Fourier series of order $N_{l}$. $\Phi =\{a_{1,1},b_{1,1},...,a_{N_{l},l},b_{N_{l},l}\}$ are the parameters that need to be trained. Event terms are constructed by setting the impact parameter κ to be applied to any data that falls within the collection D of special events (Eq. 4), which can also be flexibly adapted in form based on prior knowledge to capture more complex dynamic irregularities. The residual term is considered the component excluding the first three terms, which should follow a normal distribution with a minimised mean. The measured in-sewer data can be used directly as training data for model parameter training.

Anomaly detection is based on the k-sigma principle. The main theory of the k-sigma algorithm is to consider that the magnitude of the residual term of data is satisfied by a normal distribution $\varepsilon \left(t\right)∼normal\left(\mu ,\sigma^{2}\right)$, where μ refers to the mean value and σ represents the standard deviation. In other words, it is considered that most of the residual data should fall within the interval (μ−kσ,μ+kσ). The data outside this interval are small probability events and are considered anomalies (Fig. S1). The value k is usually chosen from the range [1,5]. The smaller the value, the more anomalies will be screened out in a single iteration, and the faster the loop (discussed in a later paragraph) will end, but too small a value may lead to misclassification of normal cases.

Since the reconstruction process of BWF is affected by the training samples, a loop iteration is used to continuously remove the anomalies according to the anomaly detection criteria for re-training, which is used to eliminate the impact of the I/I-influenced data points on the accuracy of the BWF reconstruction. The algorithm can be terminated if any one of the following criteria is satisfied:

(1). The residual term satisfies the Anderson-Darling (Nelson, 1998) normality test, which means the residual meets normalisation requirements.
(2). The maximum value of the residual term is less than a selected threshold.
(3). Correlation between two successive reconstructed BWFs above the selected threshold. Here, the Pearson coefficient can be chosen to measure the correlation. The high correlation between the two successive reconstruction results means that data point removal will no longer affect the reconstruction results (SI shows more details and equations of those three termination conditions).

The algorithm considers all the data determined as anomalies to belong to the wet conditions, and the data determined as normal are considered to belong to the dry conditions, thus identifying I/I periods. For concise expressions, here we refer to the identified periods with and without I/I as wet and dry periods/conditions, respectively, regardless of their causes (rainfall, snowmelt, or seawater tide). Hereafter, we will use these terms interchangeably depending on the context.

Based on the k-sigma principle, the anomaly detection process considers each data point independently. This may result in isolated dry points being identified within a continuous wet period or vice versa. However, given the characteristics of I/I, the periods of dry or wet conditions typically occur in longer, continuous segments. Therefore, after completing the iterations, a process can be applied to remove these isolated points. Specifically, a sliding window of several hours is used to perform majority voting for each time point. If the majority of points within the window are dry but the central point is wet, the wet point is corrected to dry, and vice versa.

This method eliminates the dependence on external data and manual pre-processing procedures. The algorithm can handle the I/I from various factors, such as rainfalls, snowmelts, and seawater tides, without any separating analysis.

### 2.2 Accuracy validation and comparison with other methods

Three other existing approaches, labelled as the Manual 1, Manual 2 and Manual 3 methods, are compared for accuracy with the newly proposed method, labelled as the Auto method. With Manual 1 (Ge et al. 2024), the 12h / 24h / 48 period following rainfalls exceeding 0.1mm, 1mm or 3mm per 5min is considered a wet period. With Manual 2 (Rezaee et al. 2022), if any 6-hour period in a day has more than 0.3mm of rainfall, that day, along with the following half day, is classified as a wet period. Manual 3 (Staufer et al., 2012) defines a wet period as having no more than 3mm of rainfall per 24 hours. If the interval between two detected rainfall periods, according to this standard, is less than 4 hours, the intervening period will also be considered in wet conditions.

We first tested the performance of the proposed algorithm, along with the three manual methods, using simulated data produced for a real sewer network (SI, Ge et al., 2024). Simulated data were used initially because knowledge of the ground truth allowed accurate determination of various metrics. We generated simulation data (Fig. S8, S9, S10) for three different scenarios (Section 4.1), each for three months, to verify the performance of the methods under different conditions. The three scenarios are a dry weather-dominated scenario, a 50/50 wet/dry weather scenario, and a wet weather-dominated scenario. Each data point (one every five minutes) was considered an independent evaluation sample, and performance statistics were performed using the binary (dry vs. wet) classification judging criteria confusion matrix, accuracy, precision, recall, and F1-Score (Section 4.3).

**Fig. 2:**
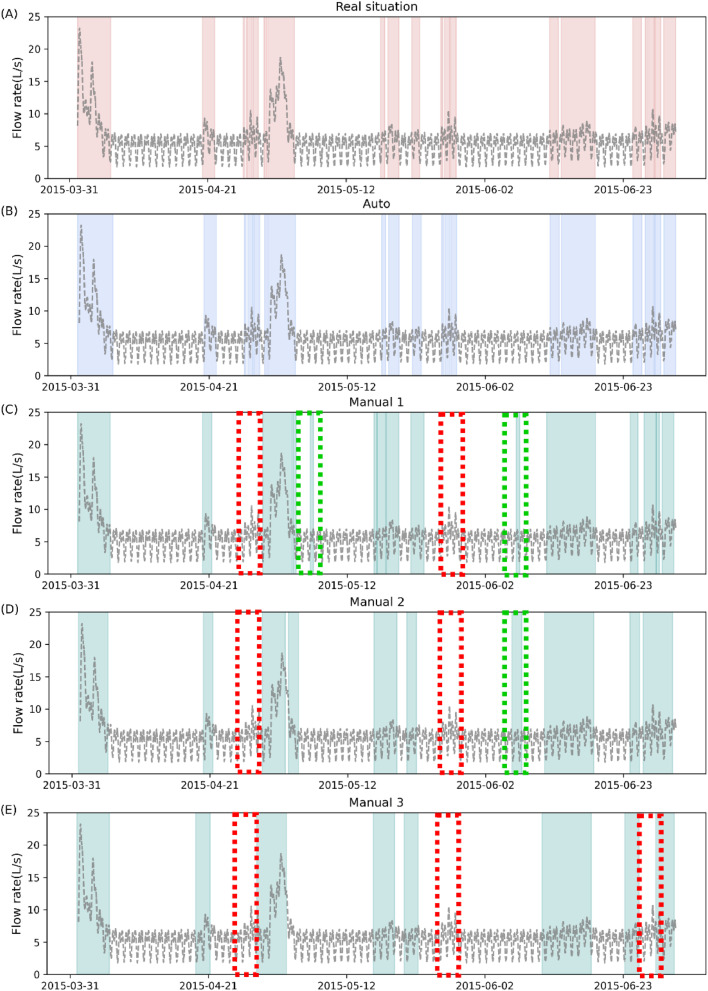
Comparison of actual and detected wet periods by various methods under the dry weather-dominated scenario. (A) The real situation of the wet periods (B) Detected wet periods using the Auto method. (C) Detected wet periods using the Manual 1 method. (D) Detected wet periods using the Manual 2 method. (E) Detected wet periods using the Manual 3 method. The grey dashed line represents the measured flow rate data; the shaded area in each subfigure represents the real wet periods and the wet periods detected by each method; the green box highlights the false detections; the red box highlights the missed detections.

By comparing the results detected by different methods as well as the real situation, the novel method is more capable of accurately determining the beginning and end of each wet period (Fig. 2, Fig. S14 and Fig. S15). There are significant advantages in dealing with the delay between rainfalls and the actual occurrence of an I/I, as well as in detecting durations. Taking the I/I event on 2015-04-21(Fig. 2) as an example, the Auto method accurately identified the start and end time in the wet period with an error of 0 and 5 minutes, respectively. In comparison, the errors in the start and end time with other methods ranged between 5 and 27 hours. In addition, the Auto algorithm was able to circumvent false detections by other methods, where rainfalls did not induce I/I, as well as missed detections of I/I that are not caused by rainfalls (Fig. 2).

**Fig. 3:**
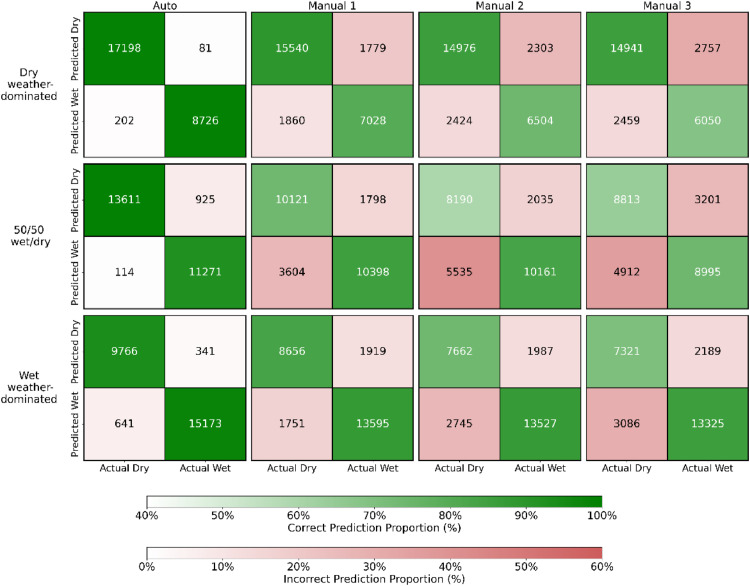
The confusion matrices of four methods (Auto, Manual 1, Manual 2, and Manual 3) applied to three scenarios: dry weather-dominated, 50/50 wet/dry, and wet weather-dominated. Each confusion matrix represents the classification results for predicted wet and dry conditions. Correct predictions (dry as dry, wet as wet) are shown in green, while incorrect predictions (dry as wet, wet as dry) are highlighted in red. The intensity of the colours indicates the proportion of correct or incorrect predictions. Numerical values represent the count of data points (with a sampling interval of five minutes).

In all scenarios, the anomaly detection-based (Auto) method outperforms existing methods (Manual 1, Manual 2, Manual 3) in all performance metrics (Fig. 3, Fig. 2, Fig. S13, Fig. S14 and Tab. 1), and the performance can reach more than 90%. This advantage is especially obvious when the number of points in wet and dry conditions is roughly equal.

**Table 1:** Comparison of different dry/wet distinguishing methods under various scenarios.

Scenario	Metrics	Method	Performance
Dry weather dominates	Accuracy	Auto	98.92 %
		Manual 1	86.11 %
		Manual 2	81.96 %
		Manual 3	80.09 %
	Precision	Auto	99.53 %
		Manual 1	89.72 %
		Manual 2	86.67 %
		Manual 3	84.42 %
	Recall	Auto	98.83 %
		Manual 1	89.31 %
		Manual 2	86.07 %
		Manual 3	85.87 %
	F1-Score	Auto	0.99
		Manual 1	0.90
		Manual 2	0.86
		Manual 3	0.85
Roughly 50/50 dry/wet weather	Accuracy	Auto	95.99 %
		Manual 1	79.16 %
		Manual 2	70.80 %
		Manual 3	68.70 %
	Precision	Auto	93.64 %
		Manual 1	84.91 %
		Manual 2	80.09 %
		Manual 3	73.36 %
	Recall	Auto	99.17 %
		Manual 1	73.74 %
		Manual 2	59.67 %
		Manual 3	64.21 %
	F1-Score	Auto	0.96
		Manual 1	0.79
		Manual 2	0.68
		Manual 3	0.68
Wet weather dominates	Accuracy	Auto	96.21%
		Manual 1	85.84 %
		Manual 2	81.74 %
		Manual 3	79.65 %
	Precision	Auto	96.62 %
		Manual 1	81.85 %
		Manual 2	79.41 %
		Manual 3	76.98 %
	Recall	Auto	93.84 %
		Manual 1	83.17 %
		Manual 2	73.62 %
		Manual 3	70.35 %
	F1-Score	Auto	0.95
		Manual 1	0.83
		Manual 2	0.76
		Manual 3	0.74

### 2.3 Performance analysis with various measured variables

In addition to flow, other measured variables, such as temperature and conductivity, have also been used to identify I/I in sewers (Zhou et al., 2023; Zhang et al., 2018b). This is because wastewater, surface water (source of inflow), and groundwater (source of infiltration) can have significantly different temperatures and conductivities. Here, we compare the performance of the proposed Auto method with flow, temperature, conductivity, and combined temperature and conductivity data as inputs. The flow, temperature and conductivity data (Fig. S8, S11, S12) used for analysis in this section are generated by the same simulation system mentioned in the last section under the dry weather-dominated scenario.

The performance of the algorithm using the temperature and conductivity data is moderate to substantially lower than that achieved with the flow data as the input (Tab. 2, Fig. S15 and Fig. S16). This is mainly due to two reasons. First, the temperature and conductivity profiles are more complex than those of flow (Fig. S8, Fig. S11, Fig. S12) because they are affected by more factors such as atmospheric temperature, geological environment, etc., making it more challenging to reconstruct the BWFs. Secondly, the effect of I/I on the flow is more direct than on other measured variables, which are affected by the properties of the water resource of I/I. Different from flow data, which would deviate from the BWF pattern as soon as I/I occur, water quality parameters such as temperature and conductivity may display smaller or even no deviation from the BWF pattern when their values in wastewater, surface water and groundwater are similar.

**Table 2:** Comparison of dry/wet distinguishing using different measured variables.

Metrics	Variable	Performance
Accuracy	Flow	98.92 %
	Temperature and Conductivity	89.04 %
	Conductivity	88.18 %
	Temperature	65.27 %
Precision	Flow	99.53 %
	Temperature and Conductivity	91.43 %
	Conductivity	91.15 %
	Temperature	75.32 %
Recall	Flow	98.83 %
	Temperature and Conductivity	92.13 %
	Conductivity	91.04 %
	Temperature	70.94 %
F1-Score	Flow	0.99
	Temperature and Conductivity	0.92
	Conductivity	0.91
	Temperature	0.73

The poorest performance was obtained when the temperature data alone was used. This was caused by the inadequate variability in temperature between wastewater, surface water and groundwater (Fig. S11). Comparatively, better performance was obtained when conductivity data was used as the input, either alone or in combination with temperature, because of the relatively significant differences in conductivity between wastewater, surface water and groundwater (Fig. S12). However, this observation is not universal, and temperature may be a more suitable variable than conductivity in other cases. Therefore, combining the two variables is recommendable, considering both are relatively easy to measure. At the same time, anomaly detection by creating an anomaly score (AS) combining two or more indicators (Eq. 5) instead of using the residuals of only one variable can help to combine the advantages of different variables and thus improve performance.

(5) $$AS\left(t\right)=\left(1-\theta \right)Norm\left(\varepsilon_{T}\left(t\right)\right)+\theta Norm\left(\varepsilon_{C}\left(t\right)\right)$$

where $\varepsilon_{T}$and $\varepsilon_{C}$ refer to the residual terms of temperature and conductivity, respectively. θ∈[0,1] is the weighting factor. Norm(x) denotes the normalisation of x.

### 2.4 Application to real data

**Fig. 4:**
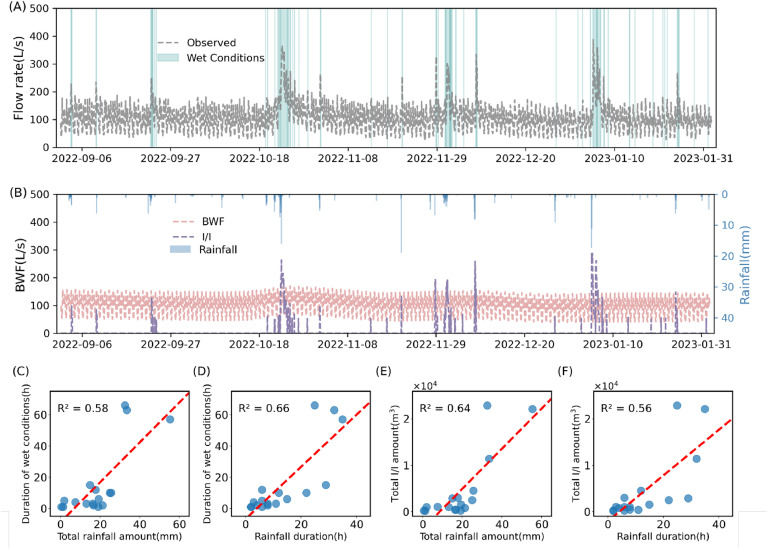
Application of the method to real-life data. (A) The flow data and the wet/dry condition distinguishing. (B) The reconstructed BWF, estimated I/I flows, and rainfall data. (C) Correlation between duration of wet conditions and total rainfall amount. (D) Correlation between duration of wet conditions and rainfall duration. (E) Correlation between the total I/I amount and total rainfall amount. (F) Correlation between the total I/I amount and rainfall duration.

Here, the algorithm has been applied to real-world flow data (5 months, hourly data). The proposed method allowed the determination of the dry/wet conditions (Fig. 4A) and the BWF and I/I flows (Fig. 4B), where I/I flows equal to the measured flow minus the reconstructed BWF. I/I generally followed rainfall events, suggesting that rainfalls are the primary cause of I/I. However, some rainfall did not induce I/I. These rainfalls were either too small to cause I/I or following extended periods of dry conditions.

The duration of wet conditions and total I/I amount determined with the proposed algorithm are strongly correlated with the total rainfall amount and duration of rainfall with an R^2^ value of 0.56 - 0.66, respectively (Fig. 4C, Fig. 4D, Fig. 4E and Fig. 4F). Considering that the rainfall data were not utilised by the algorithm, the correlations identified provide independent evidence that our algorithm correctly identified the dry/wet conditions. We applied the method to two additional real-life cases with similar performance (Fig. S17, Fig. S18).

Compared to existing manual methods, the proposed method incurs a higher computational load, particularly due to the iterative time-series reconstruction. However, the computational load remains manageable. The real-world case study reported above was conducted on a standard computational setup (Intel i7 processor, 32GB RAM). The analysis of 5-month data was completed in 2 minutes. The process could be further optimised for large datasets, by, for example, simplifying the iterative steps or leveraging parallel computing.

## 3 Conclusions

We have developed a novel method based on sewer measurements to distinguish between wet and dry conditions for sewer I/I analysis. This method uniquely leverages the theory of time series anomaly detection, which is independent of external data sources such as rainfall, a significant departure from traditional approaches that heavily rely on external information. The versatility of the method extends to its application with various in-sewer measurements, not limited to flow data. As confirmed by the simulation studies, the method significantly enhances the accuracy and precision of determining wet/dry conditions. It simplifies the process by reducing the need for extensive pre-processing and pre-analysis, typically required by conventional methods. This improvement streamlines the diagnostic process and broadens its applicability without the necessity to adjust parameters based on geographic or other environmental considerations. Moreover, the approach is robust in handling I/I caused by diverse factors beyond rainfall, including snowmelt and seawater tides. The method contributes significantly to the quantitative analysis of I/I, which is demonstrated by real-world data.

## 4 Materials and methods

### 4.1 Simulation system

The data used in 2.2, 2.3 were generated from a simulation system based on a real sewer network in a coastal city in Australia. The network includes a pumping station, 0.549 km of pressure pipeline, and 2.098 km of gravity pipeline. (For more information about the simulation system, please refer to SI.)

The simulation process includes rainfall-induced inflow and infiltration, seawater-induced infiltration, pumping station operation, and hydraulic, mass and heat transfer processes in the sewer. Using rainfall data (Fig. S5, Fig S6) from the different seasons, a dry weather dominant scenario (dry season) and a 50/50 wet/dry weather scenario (rainy season) were constructed. The wet weather dominant scenario was constructed by pooling data from multiple rainfall periods into a short period of time (Fig. S7), as the city is not in an area of perennial precipitation.

### 4.2 Data collection

The flow data used for the real-life application of the method (Section 2.4) was collected from the inlet of a wastewater treatment plant in an Australian city. The data was hourly, from September 1, 2022, to February 1, 2023. Meanwhile, for comparative analysis of the method results, rainfall data from the Bureau of Meteorology was collected at a one-minute interval.

### 4.3 Identification judging criteria

The confusion matrix, accuracy, precision, recall, and F1-Score were used to evaluate the performance of the methods with the following definitions. True dry (TD): actual dry and predicted dry; false dry (FD): actual wet and predicted dry; false wet (FW): actual dry and predicted wet; and true wet (TW): actual wet and predicted wet. Thus,

(6) $$accuracy=\frac{TD+TW}{TD+TW+FD+FW}$$

(7) $$precison=\frac{TD}{TD+FD}$$

(8) $$recall=\frac{TD}{TD+FW}$$

(9) $$F1=\frac{2precison\cdot recall}{precision+recall}$$

The accuracy, precision, recall and F1 all have values from 0 to 1, with a value closer to 1 indicating better performance.

## CRediT authorship contribution statement

**Jingyu Ge:** Writing – original draft, Visualization, Validation, Methodology, Investigation, Formal analysis, Data curation, Conceptualization. **Jiuling Li:** Writing – review & editing, Supervision, Conceptualization. **Ruihong Qiu:** Writing – review & editing, Supervision. **Tao Shi:** Writing – review & editing. **Zi Huang:** Supervision. **Yanchen Liu:** Resources. **Zhiguo Yuan:** Writing – review & editing, Supervision, Conceptualization.

## Declaration of competing interest

The authors declare that they have no known competing financial interests or personal relationships that could have appeared to influence the work reported in this paper.

## Data Availability

The authors do not have permission to share data.

## Appendix Supplementary materials

Image, application 1
