# Supplementary material for: Identifying periods impacted by sewer inflow and infiltration using time series anomaly detection
Source: Water Res X. 2024 Nov 12;25:100278. doi: 10.1016/j.wroa.2024.100278 (PMC11609673; doi:10.1016/j.wroa.2024.100278)
Supplement: Supplementary file 1 [file mmc1.docx]

**Supplementary Information**

**S1. k-sigma principle for anomaly detection**

The I/I period identification method proposed in the paper consists of an anomaly detection part for screening the wet periods based on the $k$-sigma principle. As mentioned in the paper, it is considered that most of the residual data should fall within the interval $\left( \mu-k\sigma,\mu+k\sigma\right)$. The data outside this interval are small probability events and are considered anomalies (Fig.S1).

**
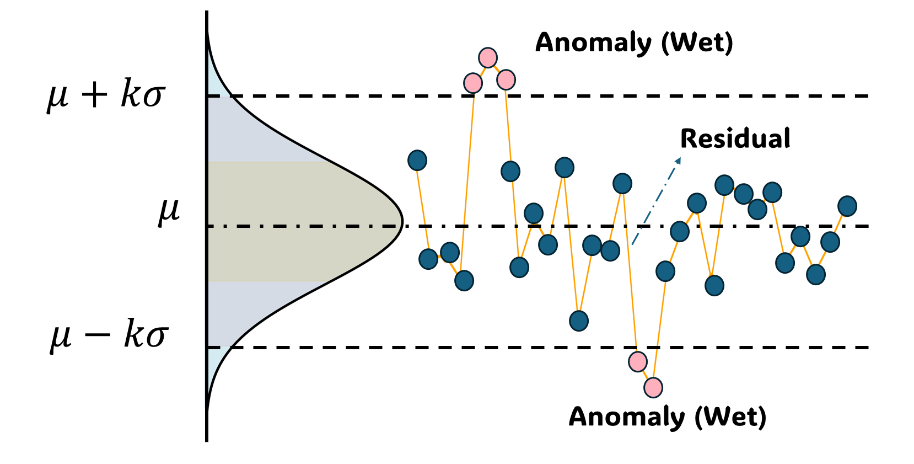
**

**Fig. S1** Schematic of the k-sigma principle for anomaly detection

**S2. Termination conditions**

The I/I period identification method proposed in the paper consists of an iteration loop algorithm in which three loop termination conditions are suggested. When the algorithm satisfies any of the three termination conditions, the algorithm can be terminated.

**S2.1 Anderson-Darling normality test**


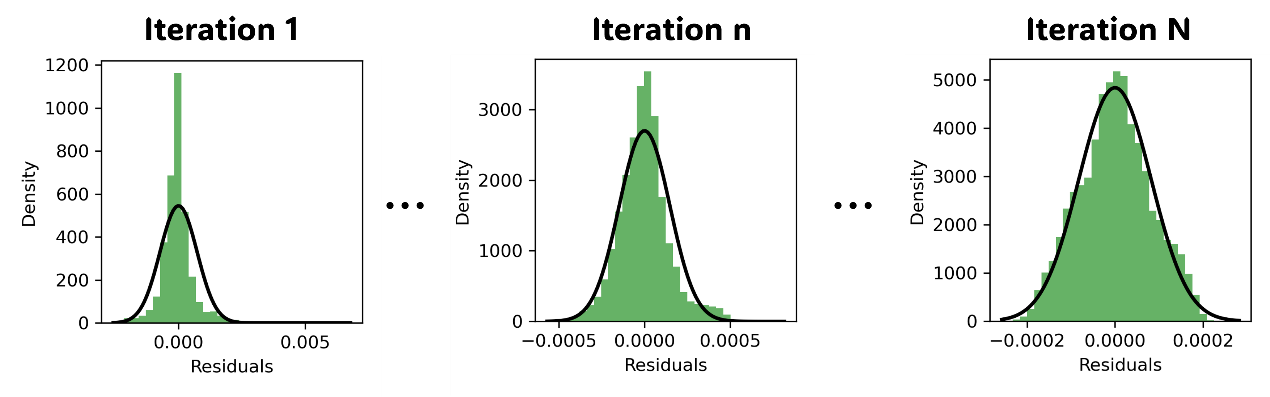


**Fig. S2** Schematic of the residual distribution with algorithm iterations

As the iterations continue, the residual sequence's distribution will converge more to a normal distribution (Fig. S2). When the residuals pass the normality test, all the anomalies (wet conditions) have been screened out.

The Anderson-Darling (AD) normality test is a commonly used method for checking whether sample data satisfy a normal distribution. The basic principle is to measure the difference between the sample data and the objective distribution by calculating the AD statistic.

Here are the steps for performing an AD test:

1. Sort the sample data in ascending order. If there are original samples $X_{1},X_{2},...,X_{n}$, then after sorting, the sorted data will be $Y_{1}\leq Y_{2}\leq...\leq Y_{n}$.
2. Calculate the mean $\mu$ and standard deviation $\sigma$ of the sample data.

$\mu=\frac{1}{n}\sum_{i=1}^{n} X_{i}$ (Eq. S1)

$\sigma=\sqrt{\frac{1}{n-1}\sum_{i=1}^{n} (X_{i}-\mu)^{2}}$ (Eq. S2)

1. Standardize the data to a standard normal distribution, which has a mean of 0 and a standard deviation of 1. Therefore, the transformed data $Z$ can be obtained.

$Z_{i}=\frac{Y_{i}-\mu}{\sigma}$ (Eq. S3)

1. For each standardised data sample $Z_{i}$, calculated the corresponding value of the cumulative distribution function (CDF) under the standard normal distribution, denoted as $F(Z_{i})$. The standard normal CDF is defined as:

$F(Z_{i})=\frac{1}{\sqrt{2\pi}}\int_{-\infty}^{Z_{i}} e^{-\frac{t^{2}}{2}}dt$ (Eq. S4)

1. Compute the AD statistic $A_{0}^{2}$.

$A_{0}^{2}=-n-\frac{1}{n}\sum_{i=1}^{n} (2i-1)[ln(F(Z_{i}))]+\ln(1-F(Z_{n+1-i}))$ (Eq. S5)

1. Adjust the$A_{0}^{2}$ for the sample size to get the final AD statistic $A^{2}$

$A^{2}=A_{0}^{2}(1+\frac{0.75}{n}+\frac{2.25}{n^{2}})$ (Eq. S6)

1. Finally, compare the $A^{2}$ with critical values. If $A^{2}$ exceeds the critical value at the given level of significance, the hypothesis that the data follows the assumed normal distribution is rejected, i.e., the loop continues; and conversely, the termination condition is satisfied, and the loop ends.

Critical values are typically provided for common significance levels such as 15%, 10%, 5%, 2.5%, and 1%, with the critical values 0.576, 0.656, 0.787, 0.918 and 1.092. These critical values have been tabulated through extensive simulations and statistical theory (Nelson, 1998).

**S2.2 Residual threshold**

As the iteration continues, the value of the residual sequence decreases (Fig. S2). When the maximum residuals are smaller than a set acceptable threshold (Fig. S3), the fitting is as expected, i.e., all wet periods are screened out.

The setting of the threshold value depends on the type of data of concern, and in general, it can be selected as 10%-20% of the measurement precision of the data. For example, for temperature data, the measurement precision of a sensor is 0.1°C, and then 0.01°C-0.02°C can be selected as the threshold value.


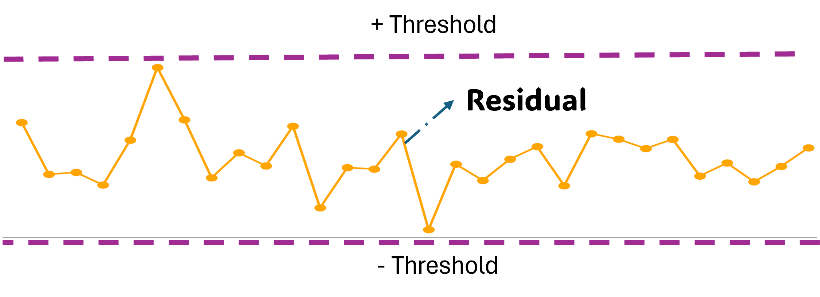


**Fig. S3** Schematic of the residual within the threshold range.

**S2.3 Correlation of two consecutive BWF reconstruction results**

As the iteration continues, anomalies will be removed so that they no longer impact the reconstruction of the BWF. Therefore, the reconstruction results of BWF will be geared towards stabilisation, i.e., the correlation between two consecutive reconstruction results will be high. When the correlation is above a certain threshold (Fig.S4), it indicates that the de-anomaly (wet conditions) has been sufficiently screened. Here, the Pearson coefficient can be chosen to measure the correlation.


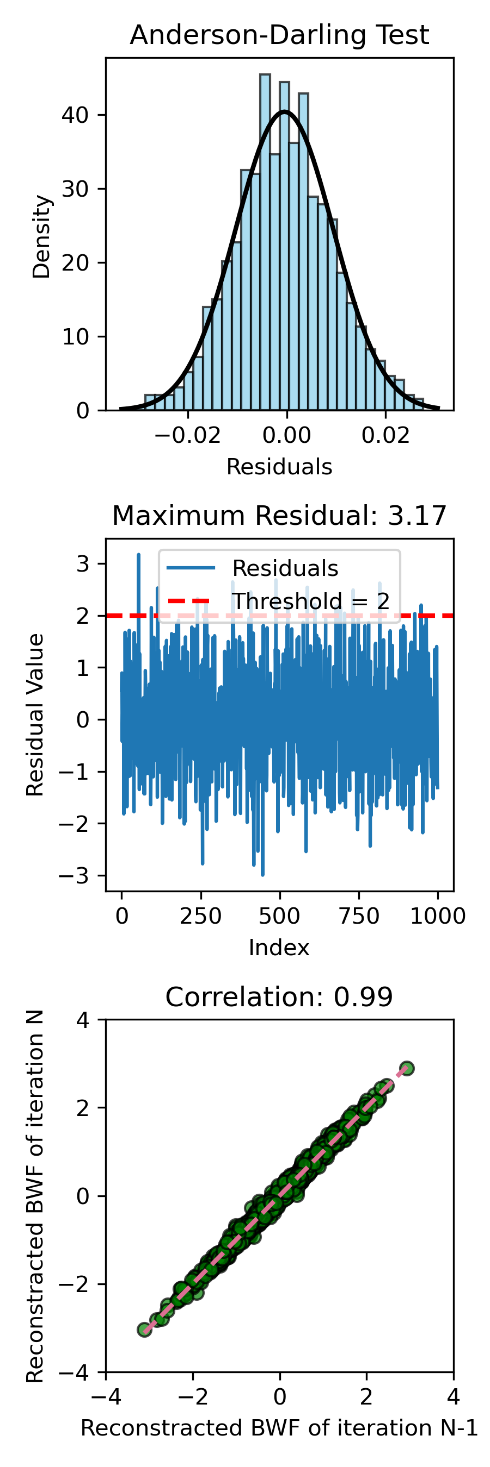


**Fig. S4** Schematic of the correlation of two consecutive BWF reconstruction

The Pearson coefficient is a statistical indicator that measures the degree of linear correlation between two variables. The closer it is to 1, the higher the correlation. ${}^{n}M$ denotes the result of the BWF reconstruction for the $n$-th iteration, and ${}^{n+1}M$denotes the results of the BWF reconstruction for the $n+1$-th iteration. The Pierce coefficient ${{}^{n+1}P}_{M}$ between those can be calculated through Eq.S7.

${{}^{n+1}P}_{M}=\frac{\sum({{}^{n}M}_{i}-\overline{{}^{n}M})({{}^{n+1}M}_{i}-\overline{{}^{n+1}M})}{\sqrt{\sum({{}^{n}M}_{i}-\overline{{}^{n}M})^{2}({{}^{n+1}M}_{i}-\overline{{}^{n+1}M})^{2}}}$ (Eq.S7)

where $\overline{{}^{n}M}$ and $\overline{{}^{n+1}M}$ refer to the mean value of ${}^{n}M$and ${}^{n+1}M$, respectively. The threshold of the correlation can be set as a value close to 1, such as 0.99, 0.993, and 0.999.

**S3. Simulation study**

This study used the same simulation system as Ge et al., 2024. For more details on the simulated models, parameters, and inputs applied in the systems, please refer to the S4 section (SI, Ge et al., 2024).

**S3.1 Simulated scenarios, simulation inputs and simulated data**

In this study, three scenarios were simulated to test the proposed method: a dry weather-dominated scenario, a 50/50 wet/dry weather scenario, and a wet weather-dominated scenario. Fig.S5, S6, and S7 display the rainfall input of the simulation system for those three scenarios.


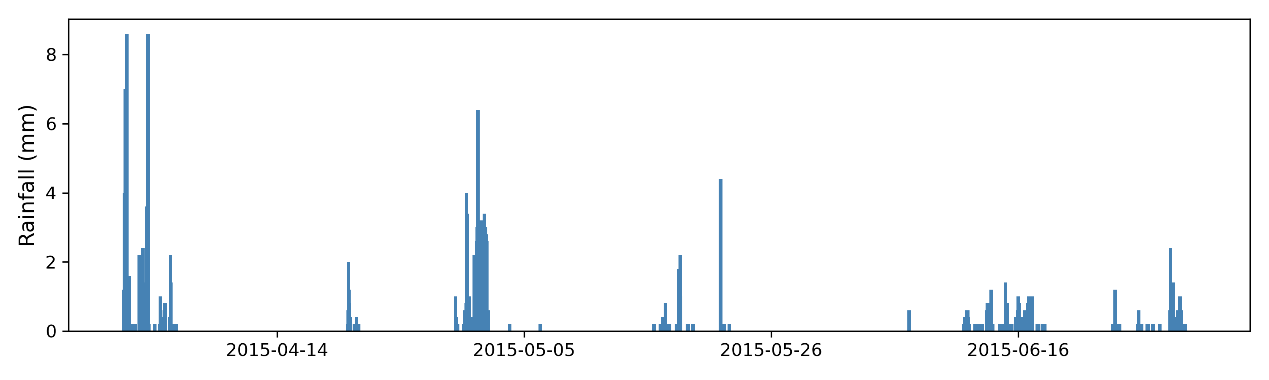


**Fig. S5** Rainfall inputs for dry weather-dominated scenario


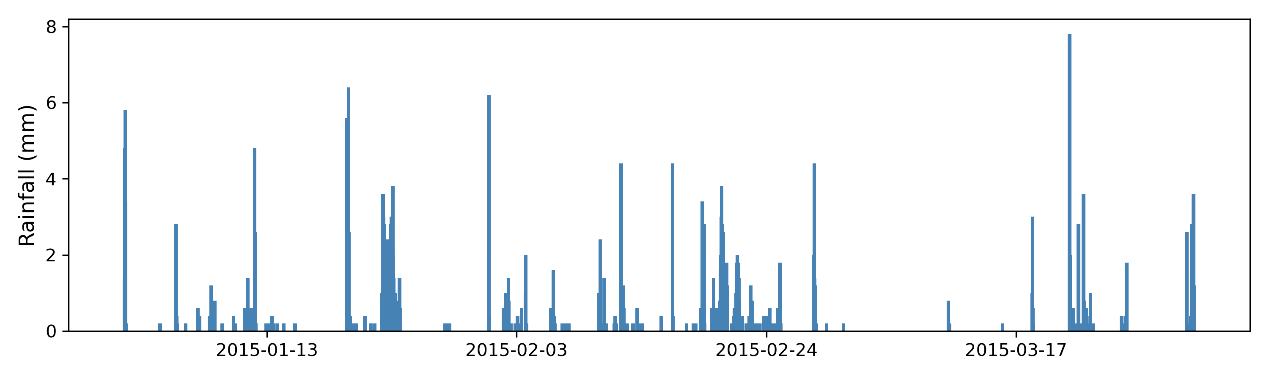


**Fig. S6** Rainfall inputs for 50/50 wet/dry weather scenario

**
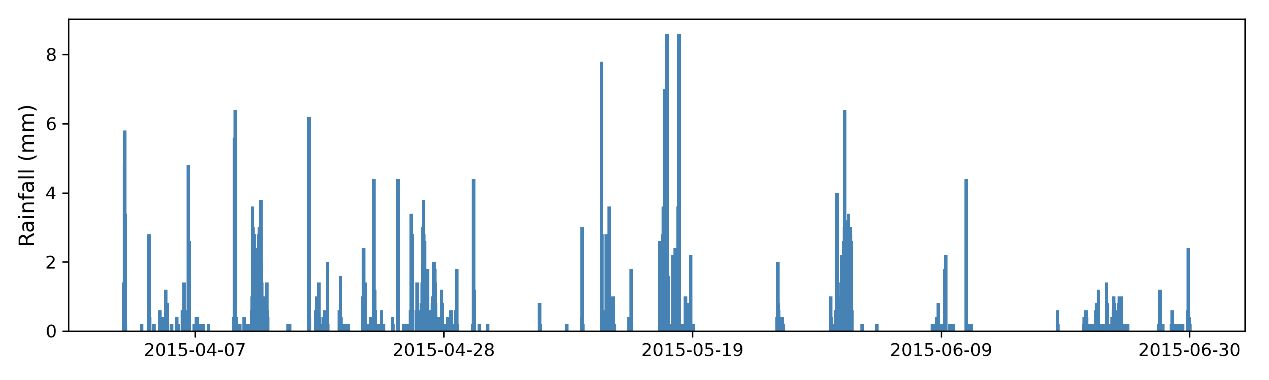
**

**Fig. S7** Rainfall inputs for wet weather-dominated scenario

Fig.S8, S9, and S10 display the generated simulation data of flow under those three scenarios. Those data are used for Section 2.2 in the paper for accuracy validation and comparison with other methods.


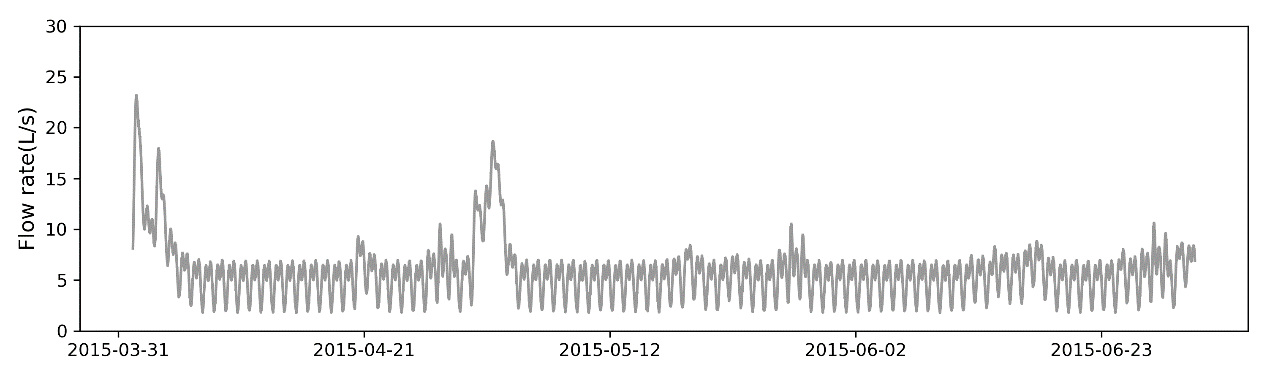


**Fig. S8** Generated flow data under the dry weather-dominated scenario


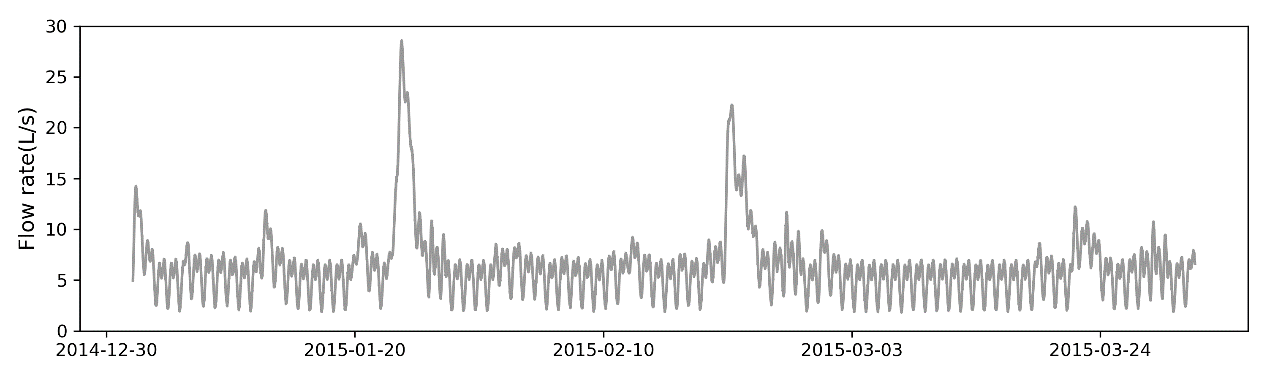


**Fig. S9** Generated flow data under the 50/50 wet/dry weather scenario


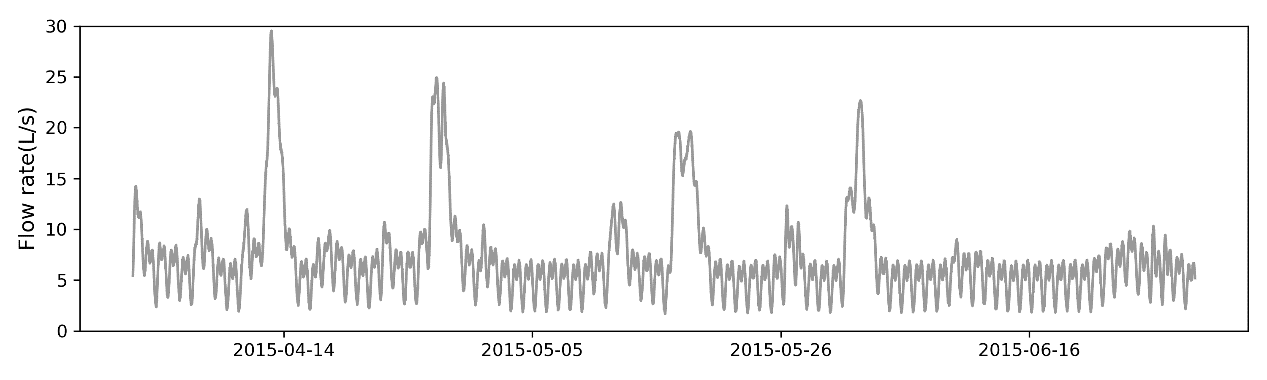


**Fig. S10** Generated flow data under the wet weather-dominated scenario

Fig.S11 and S12 display the temperature and conductivity input of the simulation system, which includes groundwater and surface water, and the simulated temperature and conductivity data, respectively. The simulated temperature and conductivity data, which are various measured variables, are used for performance analysis in Section 2.3 of the paper.


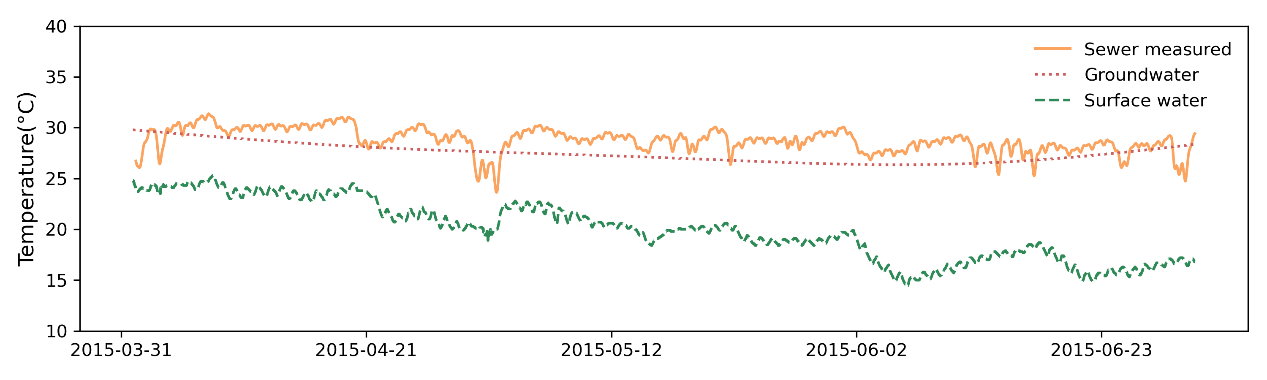


**Fig. S11** Temperature inputs of the simulation system and the simulated temperature of sewer measurement.


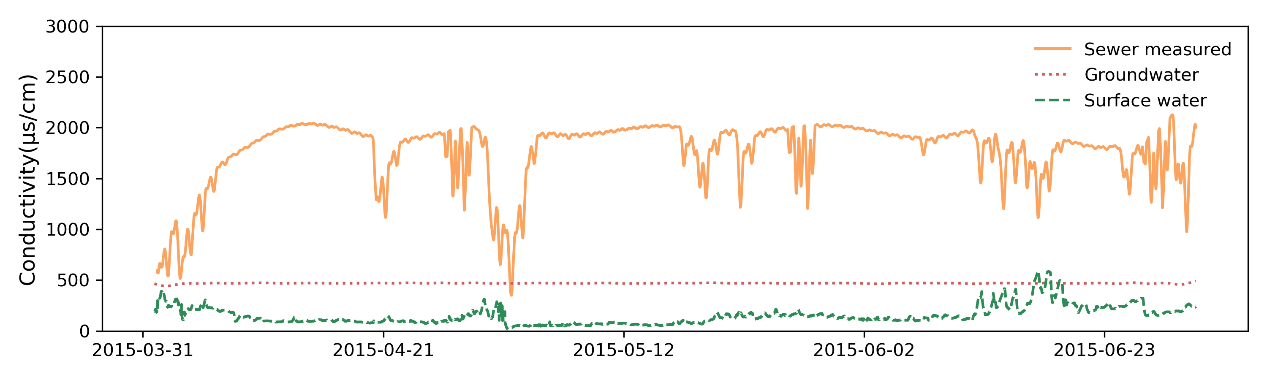


**Fig. S12** Conductivity inputs of the simulation system simulated conductivity of sewer measurement

**S4. Accuracy validation and comparison with other methods**

**
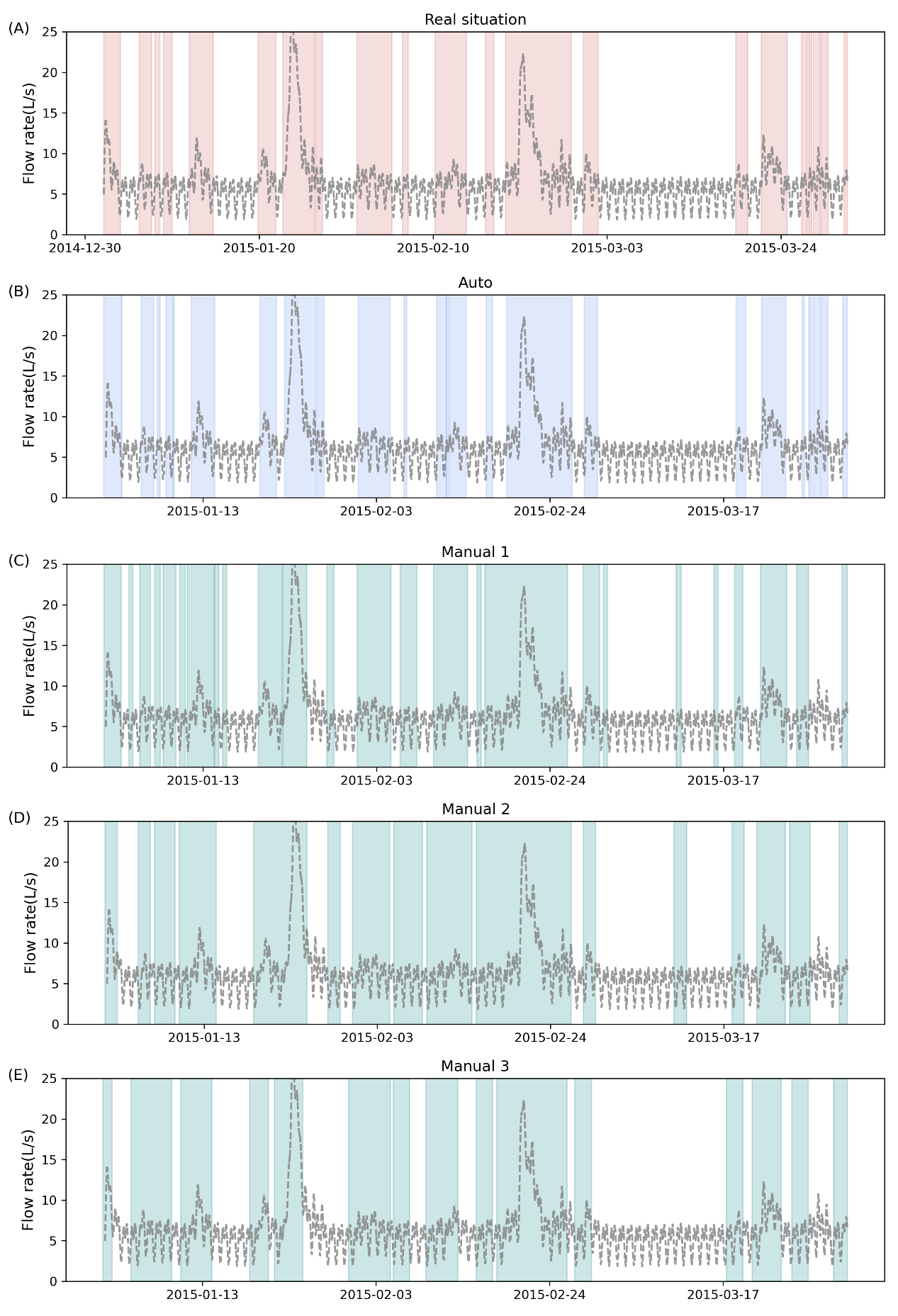
**

**Fig. S13** Comparison of actual and detected wet periods by various methods under the 50/50 wet/dry weather scenario. (A) The real situation of the wet periods (B) Detected wet periods using the Auto method. (C) Detected wet periods using the Manual 1 method. (D) Detected wet periods using the Manual 2 method. (E) Detected wet periods using the Manual 3 method. The grey dashed line represents the measured flow rate data; the shaded area in each subfigure represents the real wet periods and the wet periods detected by each method.


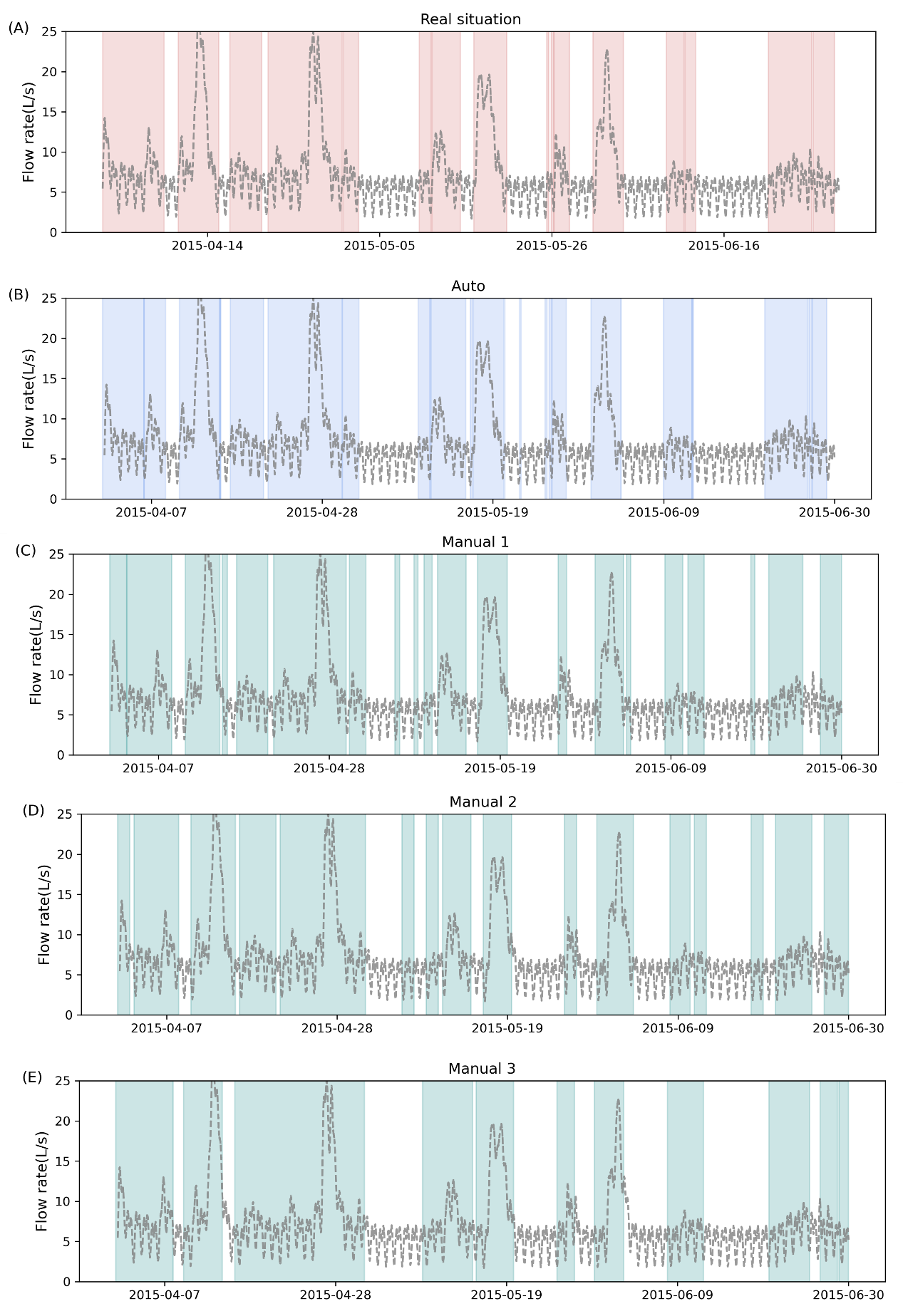


**Fig. S14** Comparison of actual and detected wet periods by various methods under the wet weather-dominated scenario (A) The real situation of the wet periods (B) Detected wet periods using the Auto method. (C) Detected wet periods using the Manual 1 method. (D) Detected wet periods using the Manual 2 method. (E) Detected wet periods using the Manual 3 method. The grey dashed line represents the measured flow rate data; the shaded area in each subfigure represents the real wet periods and the wet periods detected by each method.

**S5. Performance analysis with various measured variables**

**
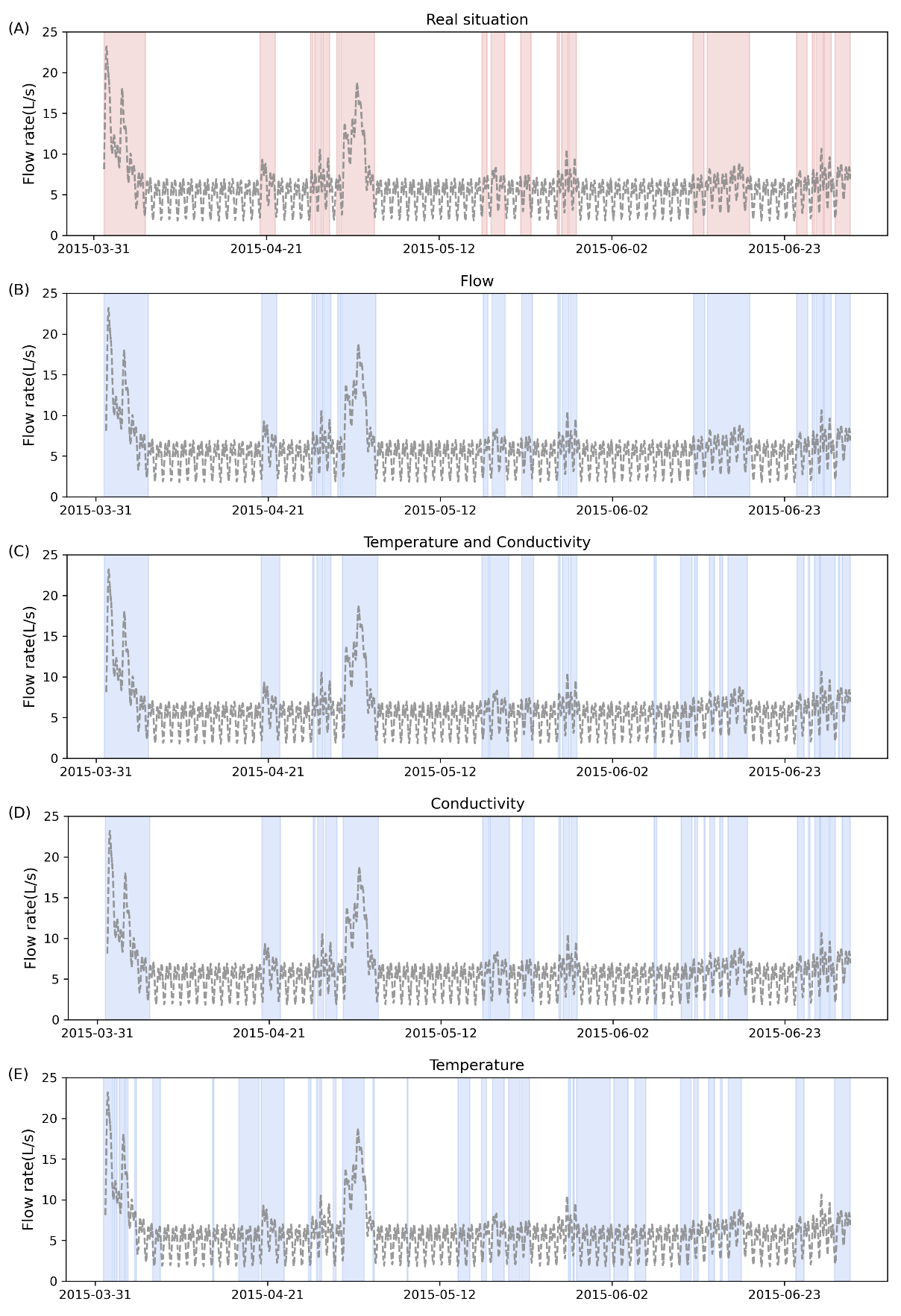
**

**Fig. S15** Comparison of actual and detected wet periods by various methods using different measured variables. (A) The real situation of the wet periods (B) Detected wet periods using Flow. (C) Detected wet periods using temperature and conductivity at the same time. (D) Detected wet periods using conductivity. (E) Detected wet periods using temperature. The grey dashed line represents the measured flow rate data; the shaded area in each subfigure represents the real wet periods and the wet periods detected by each method.


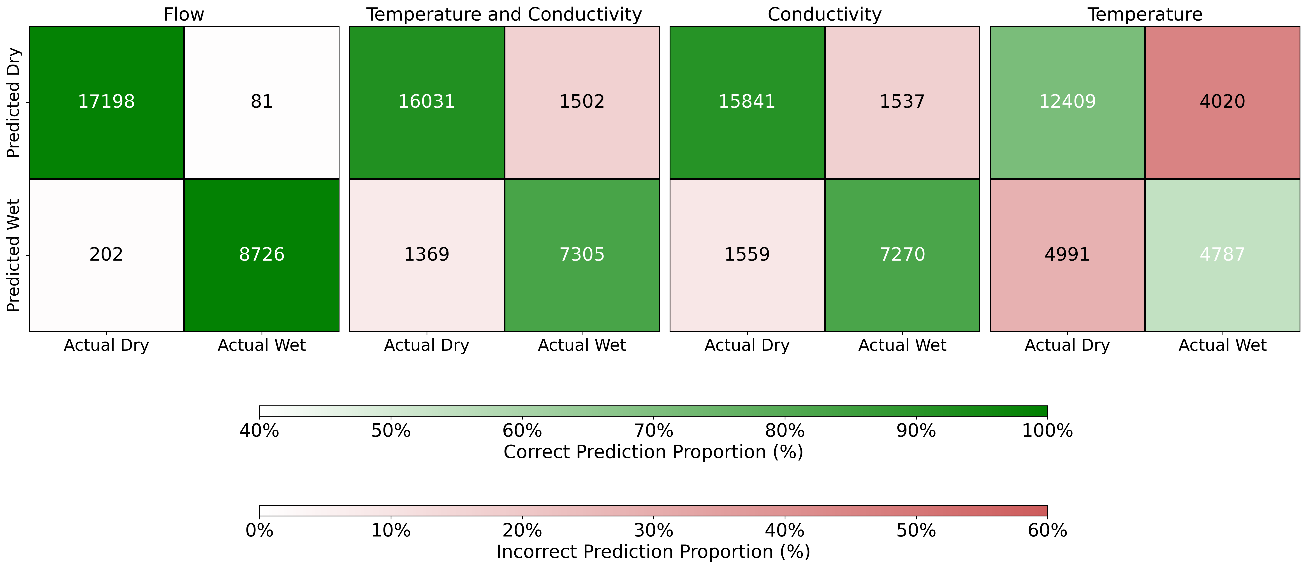


**Fig. S16** The confusion matrices of various measured variables when applied to dry weather-dominated scenarios. Correct predictions (dry as dry, wet as wet) are shown in green, while incorrect predictions (dry as wet, wet as dry) are highlighted in red. The intensity of the colours indicates the proportion of correct and incorrect predictions. Numerical values represent the count of data points (with a sampling interval of five minutes).

**S6. Two additional demonstration case studies with real data**

Two additional cases were included to test the algorithm further. Named Supplementary Case 1 (SC1) and Supplementary Case 2 (SC2), respectively, the data were collected from two cities in Australia.

SC1 contains measured flow data over 51 days, at an interval of every 15 minutes, along with corresponding rainfall data collected at the same interval. SC2 involves data from a sewer pumping station (SPS), where the sewage flow into the SPS wet well was estimated from pump operational data (SPS on/off times) and known pumped flow rates (Chen et al., 2014; Li et al., 2019) following Eq S8:

$Q_{in}=Q_{pump}\cdot\frac{(t_{2}-t_{1})}{(t_{2}-t_{0})}$ (Eq. S8)

where $Q_{in}$ refers to the average flow rate into the wet well between two pumping operations. $Q_{pump}$ is the pumped flow rate, which is a constant value. $t_{0}$ is the stop time of the previous operation. $t_{1}$ and $t_{2}$ are the start and stop time of the current operation.

This estimated flow data covers 39 days, sampled every 15 minutes, with matching rainfall data at the same frequency.

As shown in Fig.S17 and S18 (A) and (B), the algorithm successfully detects wet periods and calculates the I/I quantity. Moreover, the duration of wet conditions and the total I/I amount determined by the proposed algorithm positively correlate with the total rainfall amount and rainfall duration, as demonstrated in Fig. S17 and S18 (C), (D), (E), and (F). While these two datasets are shorter, with fewer rainfall events than the datasets in the main text, affecting the reliability of the R² values due to the limited sample size, they still demonstrate a clear and strong correlation.

**
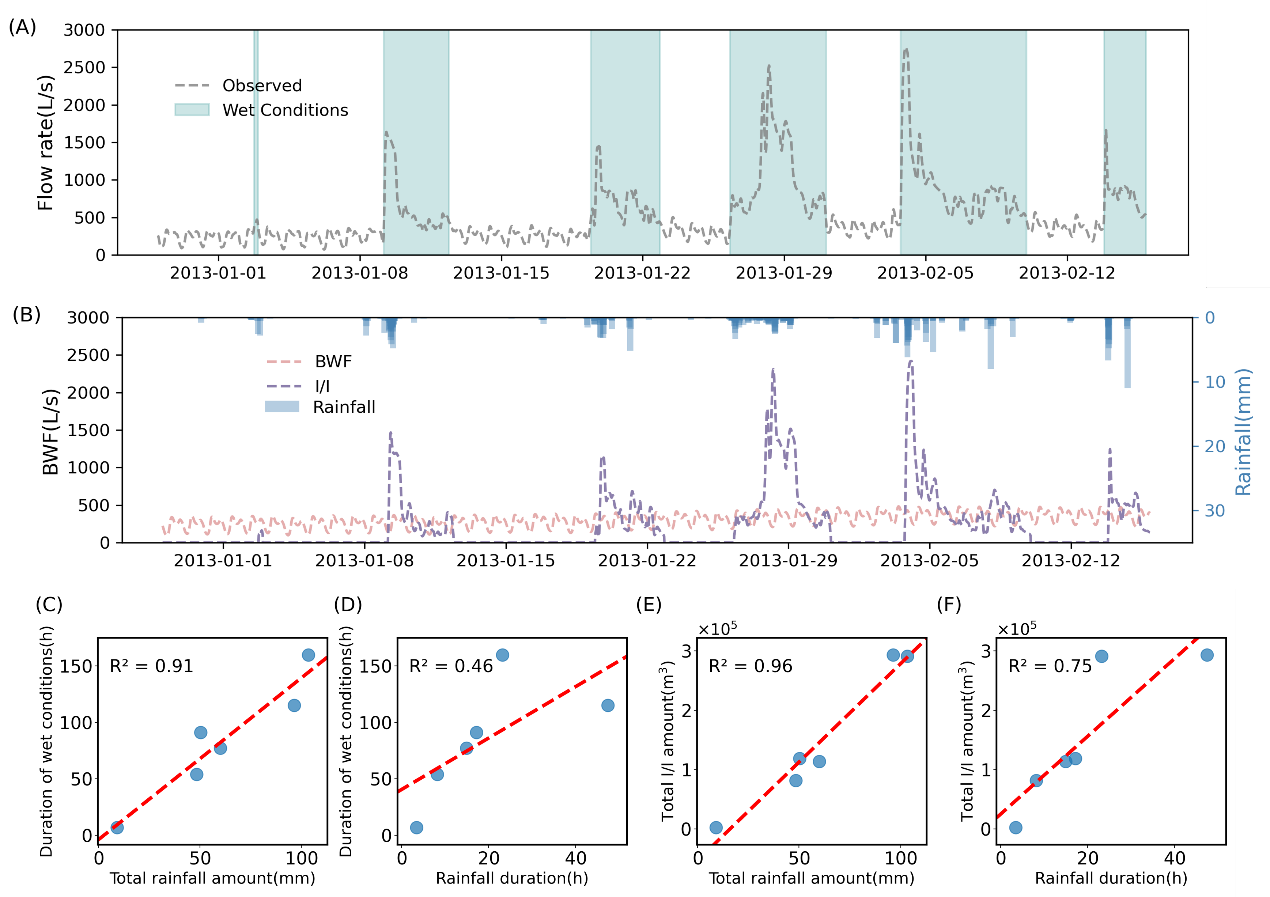
**

**Fig. S17** Application of the method to real-life data of SC1. (A) The flow data and the wet/dry condition distinguishing. (B) The reconstructed BWF, estimated I/I flows, and rainfall data. (C) Correlation between duration of wet conditions and total rainfall amount. (D) Correlation between duration of wet conditions and rainfall duration. (E) Correlation between the total I/I amount and total rainfall amount. (F) Correlation between the total I/I amount and rainfall duration.


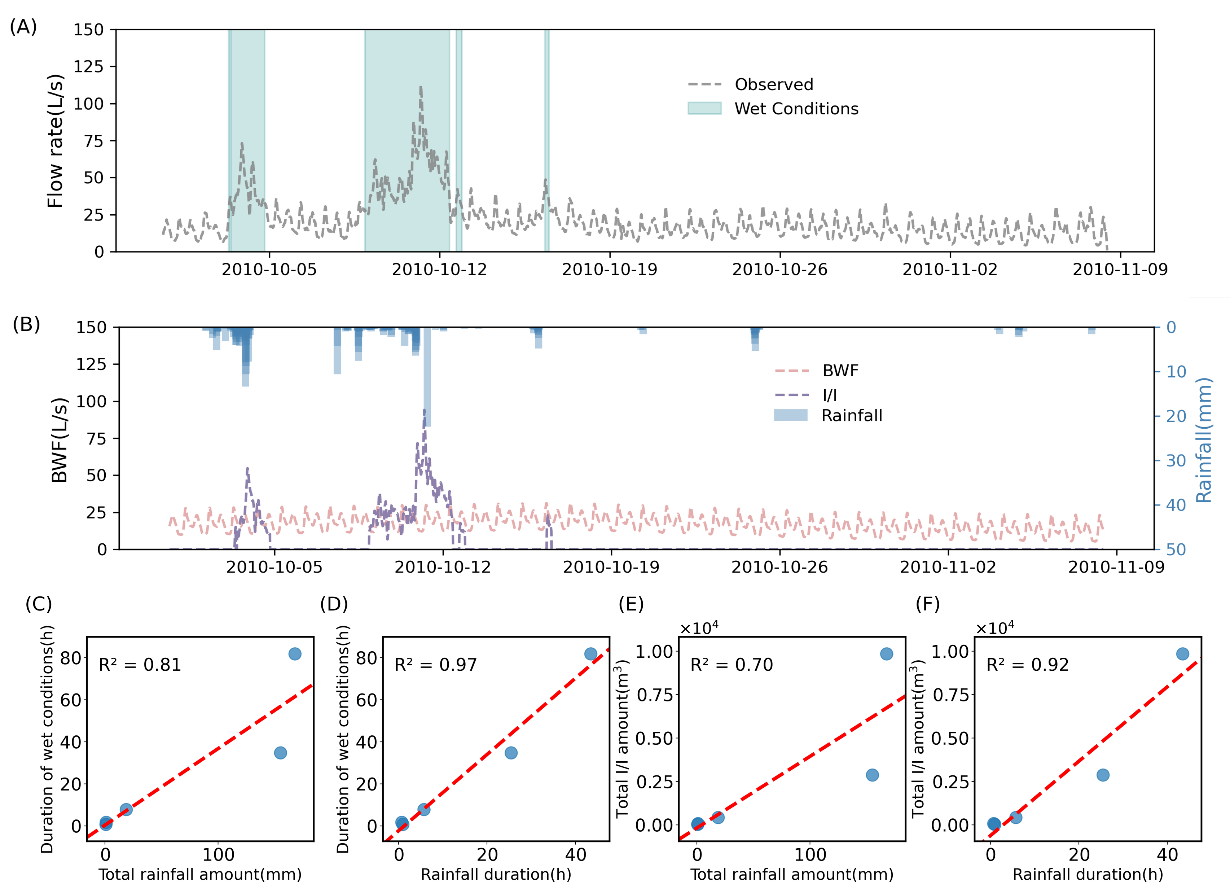


**Fig. S18** Application of the method to real-life data of sewer case SC2. (. (A) The flow data and the wet/dry condition distinguishing. (B) The reconstructed BWF, estimated I/I flows, and rainfall data. (C) Correlation between duration of wet conditions and total rainfall amount. (D) Correlation between duration of wet conditions and rainfall duration. (E) Correlation between the total I/I amount and total rainfall amount. (F) Correlation between the total I/I amount and rainfall duration.

**Reference**

Chen J, Ganigué R, Liu Y, et al. Real-time multistep prediction of sewer flow for online chemical dosing control[J]. Journal of Environmental Engineering, 2014, 140(11): 04014037.

Ge, J., Li, J., Qiu, R., Shi, T., Zhang, C., Huang, Z. and Yuan, Z., 2024. A data-driven method for estimating sewer inflow and infiltration based on temperature and conductivity monitoring. Water Research, 122002.

Li, J., Sharma, K., Liu, Y., Jiang, G. and Yuan, Z., 2019. Real-time prediction of rain-impacted sewage flow for on-line control of chemical dosing in sewers. Water Research, 149, 311-321.

Nelson, L.S., 1998. The Anderson-Darling test for normality. Journal of Quality Technology, 30(3), 298-299.
